# Cancer mortality in the indigenous population of coastal Chukotka, 1961–1990

**DOI:** 10.3402/ijch.v72i0.20471

**Published:** 2013-03-19

**Authors:** Alexey A. Dudarev, Valery S. Chupakhin, Jon Øyvind Odland

**Affiliations:** 1 Hygiene Department, Northwest Public Health Research Centre, St. Petersburg, Russia; 2 AMAP Secretariat, University of Tromsø, Tromsø, Norway

**Keywords:** Chukotka, Russian Arctic, indigenous people, Inuit, cancer mortality, epidemiology

## Abstract

**Objectives:**

The general aim was to assess the pattern and trend in cancer mortality among the indigenous people of coastal Chukotka during the period 1961–1990.

**Methods:**

All cases of cancer deaths of indigenous residents of the Chukotsky district in the north-easternmost coast of Chukotka Autonomous Okrug were copied from personal death certificates. There were a total of 219 cancer deaths during the study period. The average annual number of cases, percent, crude, and age-standardized cancer mortality rates (ASMR) per 100,000 among men and women for all sites combined and selected sites were calculated. Data were aggregated into six 5-year periods to assess temporal trends. Direct age-standardization was performed with the Segi-Doll world standard population used by the International Agency for Research on Cancer.

**Results:**

The indigenous Chukchi and Eskimo people living in Chukotsky district were at higher risk of death from cancer during the 30-year period between 1961 and 1990, with ASMR among men twice that of Russia, and among women 3.5 times higher. The excess can be attributed to the extremely high mortality from oesophageal cancer and lung cancer.

**Conclusions:**

The indigenous people of coastal Chukotka were at very high risk of death from cancer relative to the Russian population nationally. The mortality data from this study correspond to the pattern of incidence reported among other indigenous people of the Russian Arctic. Little information is available since 1990, and the feasibility of ethnic-specific health data is now severely limited.

Few studies have been conducted on the epidemiology of cancer in Chukotka (or the Russian Arctic in general) due to the remote location and sparsely distributed population, lack of ethnic identification of indigenous people, underdevelopment of oncological services, uncertain quality and completeness of diagnoses, registration and reporting, and the absence of cancer registries in any of the Russian Arctic regions.

Very high levels of cancer mortality among the indigenous people in the Russian Arctic (including Chukotka) during the 1960s and 70s (at least twice as high as in the USSR), especially of oesophageal cancer (up to 50% of the total number of cancers or more than 100 per 100,000), have long been noted (1–6). There was concern over the carcinogenicity of ionizing radiation from nuclear fallouts from atmospheric and ground explosions of atomic devices in Novaya Zemlya. However, no effect of the enhanced body burden of radiation among reindeer-herders on cancer incidence has ever been found.

A study of cancer incidence was conducted among 1,300 Chukotka Eskimos by the Russian Academy of Medical Sciences’ Institute of Internal Medicine in Novosibirsk for the period 1970–1989. There was a predominance of oesophagus (25%), lung (23%), and stomach (20%); in contrast, no cases were observed of the salivary gland, nasopharyngeal, and cervical cancers common in other Inuit populations (7). No information on mortality was provided in that article.

A study of the indigenous people of the Far East of Russia (including the Arctic territories) carried out by Khabarovsk Medical University in cooperation with the Moscow Oncological Research Centre of the Russian Academy of Medical Sciences for the period 1977–1988 shows high risk of cancer particularly among the Chukchi (all sites ASIR were 544 per 100,000 among men and 343 among women); among all studied ethnic groups the overall proportions of specific cancers were: among men – lung 29%, stomach 25%, and oesophagus 19%; among women – stomach 18%, lung 16%, cervix uteri 15%, and oesophagus 12%. The risk of oesophagus cancer was 8 times higher among indigenous men and 17 times higher among indigenous women compared to non-indigenous men and women respectively (8).

The present study was undertaken to partially fill the gap in information about cancer mortality among the indigenous people of Chukotka. The period 1961–1990 was chosen as it was a time of relatively stable Soviet health care and statistical reporting in the region. In 1991 the USSR collapsed, accompanied by major disruption of government services. Furthermore, beginning from 2002, statistical data specific to indigenous people were no longer collected. Although the data are over two decades old, they nevertheless are of historical interest, and contribute to comparison of cancer patterns among circumpolar regions and populations.

## Materials and methods

The Chukotsky district located in the north-easternmost coast of Chukotka Okrug was chosen for this study. The district's borders did not change over decades; it is inhabited by indigenous Eskimo and coastal Chukchi whose traditional livelihood has been sea mammal hunting.

Information on cancer mortality was collected from death certificates kept in the Civilian Status Registry of Lavrentiya, capital of Chukotsky *rayon* (district) for the period 1961–1990. Statistics on cancer incidence in the whole of Chukotka Autonomous Okrug is maintained by the oncological hospital in Anadyr, which is not broken down to the district level.

Primary data collection took place over several medical expeditions to Chukotka between 1970 and 1995. Cases of cancer deaths among indigenous people registered as residents of Chukotsky district were copied from personal death certificates and entered into a database. The database consists of the name of settlement, gender, age, date of death, cause of death, and cancer site. The diagnosis of cancer was not verified.

The causes of cancer death were coded in 2010 by one of us (AAD) according to the 10th revision of the International Classification of Diseases (ICD-10), as ICD codes were not used in the death certificates. To examine temporal trends, 5-year periods were averaged. Ethnic-, age-, and sex-specific cancer mortality rates were calculated using the population data of the indigenous population resident in Chukotka district obtained from the Chukotka Okrug Statistical Bureau. Comparison data for Russia (at that time the Russian Soviet Federative Socialist Republic of the USSR) for different intervals of the 30-year period were collected from several official statistical collections (9–11). Age- and sex-specific population data were based on the all-Union censuses of 1959, 1970, 1979, and 1989.

Age-standardized cancer mortality rates (ASMR) and 95% confidence interval (CI) were calculated for each sex and cancer site using the direct method based on the Segi-Doll world standard population used by the International Agency for Research on Cancer (12). Data on five sites for men and seven sites for women are presented in this report.

Table I shows the ethnic and sex distribution of the indigenous population of Chukotsky district according to the 1970, 1979, and 1989 USSR censuses. The mean population size of all indigenous people in Chukotsky district in six 5-year periods during 1961–1990 is shown in Table II.

**Table I:** Ethnic structure and population number of indigenous people of Chukotsky district

	1970		1979		1989	
			
	*N*	%	*N*	%	*N*	%
Indigenous	2678		2952		3432	
Chukchi	2384	89.0	2620	88.8	3067	89.4
Eskimo	290	10.8	323	10.9	340	9.9
Evens	4	0.1	7	0.2	15	0.4
Male	1282		1397		1668	
Chukchi	1151	89.8	1242	88.9	1491	89.4
Eskimo	131	10.2	151	10.8	168	10.1
Evens	0	0	3	0.2	4	0.2
Female	1396		1555		1764	
Chukchi	1233	88.3	1378	88.6	1576	89.3
Eskimo	159	11.4	172	11.1	172	9.8
Evens	4	0.3	4	0.3	11	0.6

**Table II:** Number of indigenous people of Chukotsky district in 1961–1990, averaged for 5-year periods

Years	Male	Female	M+F
1961–65	1,229	1,287	2,519
1966–70	1,261	1,367	2,627
1971–75	1,318	1,462	2,780
1976–80	1,376	1,565	2,941
1981–85	1,499	1,665	3,164
1986–90	1,631	1,775	3,406

During 1961–1990 10% of all cancer deaths a total of 219 cancer deaths registered in Chukotsky district were among indigenous people – 106 men and 113 women, and 197 Chukchi and 22 Eskimo.

## Results

Tables III and IV present the number of cancer deaths and mortality rates by site in Chukotsky district (ChD) compared to the whole of Russia in the 1961–1990 period among men and women. For all sites combined, the ASMR for ChD was 417 per 100,000 (95% CI: 332–501) among men and 359 per 100,000 (95% CI: 277–440) among women. The male rate was twice the ASMR among Russian men (207 per 100,000; 95% CI: 195–211) while the female rate was 3.5 times the ASMR among Russian women (98 per 100,000: 95% CI: 95–109).

**Table III:** Average annual number of cases, percent, crude and age-standardized cancer MORTALITY rates (ASMR) per 100,000 among men in Chukotsky district, 1961–1990 (averaged for 30 years)

	Chukotsky district					Russia				
		
Male	Cases/year	%	Crude	ASMR	95% CI	Cases/year	%	Crude	ASMR	95% CI
Oral cavity	0.13	3.8	9.9	11.6	0–26.1					
Pharynx	0.03	0.9	2.7	3.0	0–10.6	4495.7	3.4	6.8	6.9	4.9–7.1
Oesophagus	0.77	21.7	56.9	110.9	50.7–171.1	5043.6	4.0	7.7	8.4	7.8–8.4
Stomach	0.53	15.1	38.3	58.3	32–84.5	30592.1	24.9	47.1	51.3	46–76.4
Intestine	0.17	4.7	11.5	18.3	1.1–35.5					
Liver	0.13	3.8	10.4	23.9	0–67.8					
Pancreas	0.07	1.9	4.6	8.6	0–23.8					
Lung/trachea/bronchi	1.00	28.3	68.9	106.2	349–177.6	40122.4	31.3	61.0	64.4	50.7–66.9
Bones/cartilages	0.17	4.7	11.7	15.2	11–29.3					
Kidneys	0.00	0.0	0.0	0.0	0–0					
Urinary bladder	0.07	1.9	5.0	5.8	0–15.2					
Brain/CNS	0.07	1.9	5.3	5.7	0–14.9					
Lymphoid/HT	0.07	1.9	4.8	9.2	0–25.6	6175.4	4.8	9.4	9.8	9.3–10.4
Other sites	0.33	9.4	24.6	40.1	0–95.2					
All sites	3.53	100.0	254.7	416.7	332.2–501.1	126765	100.0	194.2	207.3	195.4–210.5

CNS=central nervous system; HT=hematopoietic tissues.

**Table IV:** Average annual number of cases, percent, crude and age-standardized cancer MORTALITY rates (ASMR) per 100,000 among women in Chukotsky district, 1961–1990 (averaged for 30 years)

	Chukotsky district					Russia				
		
Female	Cases/year	%	Crude	ASMR	95% CI	Cases/year	%	Crude	ASMR	95% CI
Oral cavity	0.07	1.8	4.5	7.0	0–19.4					
Pharynx	0.03	0.9	2.5	8.1	0–29.1	336.5	0.3	0.4	0.3	0.3–0.3
Oesophagus	0.73	19.5	49.9	82.7	15.3–150.1	3000.1	2.7	4.0	2.4	1.9–3.7
Stomach	0.57	15.0	38.0	64.5	30.1–98.8	27127.7	24.4	36.0	22.8	19.9–35.8
Intestine	0.13	3.5	8.0	9.1	0–22.3					
Liver	0.27	7.1	18.5	23.1	3–43.2					
Pancreas	0.00	0.0	0.0	0.0	0–0					
Lung/trachea/bronchi	0.87	23.0	54.0	68.6	13.9–123.3	8266.2	7.3	10.9	6.9	6.5–7.1
Bones/cartilages	0.20	5.3	13.4	16.2	5.7–26.7					
Breast	0.10	2.7	6.5	11.2	0–25	12440.7	10.9	16.3	11.6	6.3–12.7
Corpus/cervix uteri	0.33	8.8	22.2	26.7	0–54.8					
Cervix uteri						6652.9	5.9	8.8	6.0	5.6–8
Ovary	0.10	2.7	6.9	6.8	0–14.6					
Kidneys	0.03	0.9	2.5	8.1	0–29.1					
Urinary bladder	0.03	0.9	1.9	2.5	0–8.9					
Brain/CNS	0.07	1.8	4.0	3.9	0–10.2					
Lymphoid/HT	0.03	0.9	2.0	2.7	0–9.5	5608.1	4.9	7.4	5.8	5.7–6.1
Other sites	0.20	5.2	13.1	17.5	4.8–30.1					
All sites	3.77	100.0	248.0	358.6	276.9–440.3	113053.5	100.0	149.4	98.4	95.3–108.6

CNS=central nervous system; HT=hematopoietic tissues.

The leading causes of cancer death in ChD among both men and women were lung/trachea/bronchi (28% among men and 23% among women), followed by oesophagus (22 and 20% respectively) and stomach (15% each). Among men, the ASMR for cancer of the oesophagus was 111 per 100,000 (95% CI: 51–171), 13 times the Russian rate of 8.4 per 100,000 (95% CI: 7.8–8.4); similarly among women, the ASMR for oesophageal cancer in ChD, 83 per 100,000 (95% CI: 15–150), was 35 times the Russian rate of 2.4 per 100,000 (95% CI: 1.9–3.7). There was an excess of mortality for lung cancer also among ChD women, with a rate of 69 per 100,000 (95% CI: 14–123), almost 10 times the Russian rate of 7 per 100,000 (95% CI: 6.5–7.1). While the male ChD rate was very high (106 per 100,000; 95% CI: 35–178), the Russian rate was also quite high (64 per 100,000; 95% CI: 51–67) and the difference is not statistically significant.

**Fig. 1:**
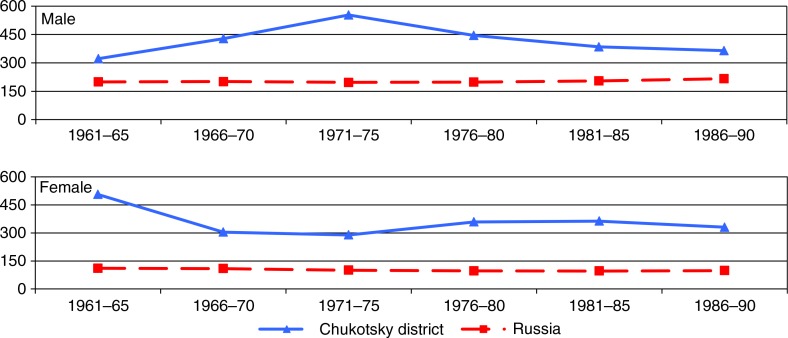
Age-standardized mortality rates (ASMR per 100,000), all cancer sites, among men and women in Chukotsky district and Russia, 1961–1990.

The rate of cancer mortality (ASMR) for all sites combined in 1961–1990 (during 30-year period) in Russia was stable and much lower than that in ChD: among men by 50–150%, and among women by 200–400% (Fig. 1).

**Fig. 2:**
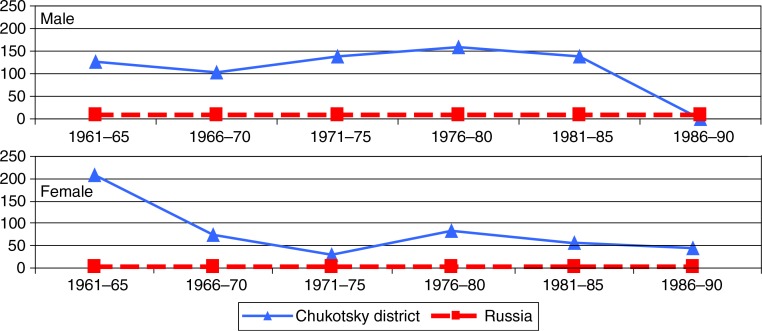
Age-standardized mortality rates (ASMR per 100,000) for oesophageal cancer among men and women in Chukotsky district and Russia, 1961–1990.

Oesophagus cancer (Fig. 2) mortality rates were consistently higher, some 12–20 times among ChD men compared to Russian men, except for the 1986–1990 period when no deaths were registered. Among women the difference was much greater (up to 50 times), but the difference appears to have narrowed since the 1960s.

**Fig. 3:**
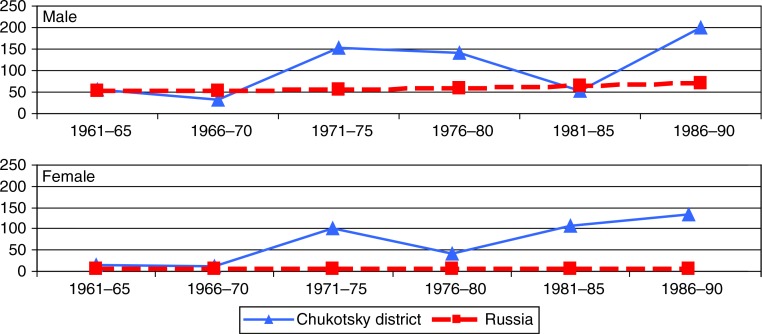
Age-standardized mortality rates (ASMR per 100,000) for cancer of the lungs, trachea and bronchi among men and women in Chukotsky district and Russia, 1961–1990.

Throughout the 30-year period, there is no clear trend in lung cancer mortality rates among ChD men, whereas among ChD women the difference was much larger – up to 20 times, with a clear increasing trend (Fig. 3).

We do not intend to discuss here the aetiology of oesophageal cancer among indigenous people of Chukotka; there is a huge amount of literature on the risk factors for oesophageal cancer from epidemiological studies in different parts of the world far from the Arctic. Dietary, behavioural, genetic, and environmental factors have been implicated, but none have been confirmed as the causative agent.

## Conclusions

The indigenous Chukchi and Eskimo people living in Chukotsky district were at higher risk of death from cancer during the 30-year period between 1961 and 1990, with ASMR among men twice that of Russia, and among women 3.5 times higher. The excess can be attributed to the extremely high mortality from oesophageal cancer and lung cancer. The pattern for mortality corresponds to that for incidence among the Eskimo in Chukotka during 1979–88 (7) and for all indigenous people in the Russian Far East and Far North during 1977–88 (8). Data specific to indigenous people since the dissolution of the Soviet Union have not been published, and unfortunately the ability to do so is no longer feasible.

## References

[1] Ramzaev PV, Troitskaia MN, Yermolajeva AP, Nizhnikov AI, Teplykh LA, Ramzaev PV (1978). Ultralinear effects of low levels of ionizing radiation. The tasks of hygienic science and practice in enhancement of effectiveness and quality of the state sanitary surveillance when controlling the use of nuclear energy in peaceful purposes. Radiation Hygiene. Collection of scientific transactions.

[2] Troitskaia MN, Yermolajeva AP, Miretskiy GI, Shubik VM, Ramzaev PV (1981). Radiation factor in the Far North and the problems of the population health. Radiation hygiene. Collection of scientific transactions.

[3] Ramzaev PV, Miretsky GI, Troitskaya MN, Doudarev AA (1993). Radiological peculiarities around Novaya Zemlja (USSR) atomic testing range. Int J Radiation Hygiene.

[4] Miretsky GI, Doudarev AA, Ramzaev PV, Troitskaya MN (1993). On the problem of mortality and oncological risk in radiation hygiene. Int J Radiation Hygiene.

[5] Miretsky GI, Doudarev AA, Popov AO, Ramzaev PV, Troitskaya MN Thirty years radiological and epidemiological monitoring in the Russian Arctic.

[6] Doudarev AA, Miretsky GI, Popov AO (1995). Radioecology and the health of the indigenous inhabitants in Chukotka. Proceedings from the International Conference on Human Health and Pollutants in the Arctic Environment. Tromso, Norway; 22–24 September 1995. J Arctic Medical Research.

[7] Nikitin YP, Boichenko NS, Astakhova TI, Dokuchaev AT, Shubnikov EV (1996). Cancer in Russian Inuit. Acta Oncol.

[8] Zaridze DG, Marochko A, Basieva T, Duffy SW (1993). Cancer incidence in the native peoples of far eastern Siberia. Int J Cancer.

[9] Napalkov NP, Tserkovniy GF, Demidova VP, Merabischvili VM (1981). Cancer mortality of the population of the USSR.

[10] Trapeznikov NN, Tserkovniy GF, Biletova BV, Dvoirin VV (1989). Malignant neoplasms in the USSR and federal republics (statistical collection). Part 2 – mortality.

[11] Chissov VI, Starinsky VV, Remennik LV (1998). Malignant neoplasms in Russia in 1980–1995.

[12] Whelan SL, Parkin DM, Ferlay J, Teppo L, Thomas DB (2002). Cancer Incidence in Five Continents.

